# Effect of the Fatigue Induced by a 110-km Ultramarathon on Tibial Impact Acceleration and Lower Leg Kinematics

**DOI:** 10.1371/journal.pone.0151687

**Published:** 2016-03-31

**Authors:** Marlene Giandolini, Philippe Gimenez, John Temesi, Pierrick J. Arnal, Vincent Martin, Thomas Rupp, Jean-Benoit Morin, Pierre Samozino, Guillaume Y. Millet

**Affiliations:** 1 Laboratory of Exercise Physiology (EA4338), University Savoie Mont Blanc, Le Bourget-du-Lac, France; 2 Laboratory Culture Sport Health Society (EA 4660), University of Franche-Comté, Besançon, France; 3 Human Performance Laboratory, Faculty of Kinesiology, University of Calgary, Calgary, Canada; 4 Laboratory of Exercise Physiology (EA4338), University of Lyon, Saint-Etienne, France; 5 Institut de Recherche Biomédicale des Armées (IRBA), Fatigue and Vigilance Team, Brétigny-sur-Orge, France; 6 Laboratoire des Adaptations Métaboliques à l’Exercice en conditions Physiologiques et Pathologiques (EA3533), Université Blaise Pascal Clermont Auvergne, Clermont-Ferrand, France; 7 Laboratory of Human Motricity, Education Sport and Health (EA6312), University of Nice Sophia Antipolis, Nice, France; University of Alabama at Birmingham, UNITED STATES

## Abstract

Ultramarathon runners are exposed to a high number of impact shocks and to severe neuromuscular fatigue. Runners may manage mechanical stress and muscle fatigue by changing their running kinematics. Our purposes were to study (i) the effects of a 110-km mountain ultramarathon (MUM) on tibial shock acceleration and lower limb kinematics, and (ii) whether kinematic changes are modulated according to the severity of neuromuscular fatigue. Twenty-three runners participated in the study. Pre- and post-MUM, neuromuscular tests were performed to assess knee extensor (KE) and plantar flexor (PF) central and peripheral fatigue, and a treadmill running bouts was completed during which step frequency, peak acceleration, median frequency and impact frequency content were measured from tibial acceleration, as well as foot-to-treadmill, tibia-to-treadmill, and ankle flexion angles at initial contact, and ankle range of motion using video analysis. Large neuromuscular fatigue, including peripheral changes and deficits in voluntary activation, was observed in KE and PF. MVC decrements of ~35% for KE and of ~28% for PF were noted. Among biomechanical variables, step frequency increased by ~2.7% and the ankle range of motion decreased by ~4.1% post-MUM. Runners adopting a non rearfoot strike pre-MUM adopted a less plantarflexed foot strike pattern post-MUM while those adopting a rearfoot strike pre-MUM tended to adopt a less dorsiflexed foot strike pattern post-MUM. Positive correlations were observed between percent changes in peripheral PF fatigue and the ankle range of motion. Peripheral PF fatigue was also significantly correlated to both percent changes in step frequency and the ankle angle at contact. This study suggests that in a fatigued state, ultratrail runners use compensatory/protective adjustments leading to a flatter foot landing and this is done in a fatigue dose-dependent manner. This strategy may aim at minimizing the overall load applied to the musculoskeletal system, including impact shock and muscle stretch.

## Introduction

The attenuation of shock waves generated at foot strike is an important function of the human musculoskeletal system and performed by bone bending, subchondral bone, intervertebral discs and heel pads [1,2]. Muscles also actively participate in shock attenuation according to the “*muscle tuning*” paradigm. This paradigm proposes that muscle activity is tuned in response to impact force characteristics to dampen soft-tissue vibrations [3,4]. This damping may occur in order to minimize detrimental effects of repetitive shocks and vibrations [5,6]. Competitive running activities induce muscle fatigue, which may impair muscle cushioning abilities [7,8,9,10,11].

The effects of fatiguing exercises on impact shock severity and attenuation have been investigated and two conflicting interpretations have been proposed. One suggests that the exercise-induced muscle fatigue lead to higher shock severity at the tibia, sacrum and/or head levels because of muscle fatigue, leading to a decreased ability to cushion impact. Numerous studies investigating short and intense running exercises found increases in peak tibia [7,8,9,10,11], sacrum [8,11], and head accelerations [12] with fatigue with increased or similar stride rates or lengths [7,8,13] compared to the non-fatigued state. Meanwhile, the frequency content of shock accelerations changes such that there is greater high frequency content at the tibia [8] and lower shock attenuation [13] after exercise. In terms of kinetics, Clansey et al. [12] found higher loading rates with fatigue.

The alternative interpretation proposes that runners strive to maintain or decrease impact magnitude by making kinematic adjustments to counteract the fatigue-induced decrease in muscle cushioning abilities. For instance, Abt et al. [14] and Clansey et al. [12] observed no change in peak tibial and head acceleration and/or shock attenuation after exercise. Also, Derrick et al. [7] found increased peak tibial acceleration but no change in peak head acceleration, thus concluding there was an overall improvement in shock attenuation after a fatiguing exercise. In terms of kinetics, lower vertical loading rates and braking forces were reported after a 5-km run at maximal intensity [15] and exhausting exercise at the maximal oxygen consumption intensity [15,16]. Likewise, vertical loading rates decreased after an extreme 8 500-km run from Paris to Beijing [17]. Several changes in kinematics and mechanics were also reported after fatiguing exercises such as greater knee flexion [7] and plantarflexion [12] at initial contact, step frequency and leg stiffness [17,18,19,20]. It was suggested that these changes could originate from a lower tolerance to repetitive shocks, especially in extreme-duration activities.

Interestingly, a “smoother and safer running style” was observed by Morin and collaborators after a 24-h treadmill run and a 166-km mountain ultramarathon (MUM) race [19,20]. This protective running style was described by an increased step frequency and a decreased downward displacement of the center of mass. Similar kinematic and mechanical adjustments were observed at the halfway point and after a 330-km MUM [21]. Based on the average stride length reported during a 5-km run [15] and during a 9.5-km hill run [22], we can estimate that over 5 km a runner would strike the ground approximately 3,000 times *versus* approximately 117,000 foot-to-ground contacts during a MUM race of ∼160 km. Combined with much greater impact shock severity running downhill [23,24], UT runners are likely subject to much greater stress applied to their joints, bones, cartilage and other musculoskeletal structures compared to middle distance track or road runners, even if their average running speed is much lower. Second, after extreme duration running exercises, large decreases in maximal voluntary contraction force, deficits in voluntary activation, muscle damage, inflammation and, to a lower extent, excitation-contraction coupling failure, occurred at both knee extensors (KE) and plantar flexors (PF) [25,26,27]. Severe lower limb muscle fatigue might further reduce the ability of the muscular system to cushion impact as mentioned above. A smoother running style might also result from ultrastructural damage induced by repetitive stretch-shortening cycles in MUM races. Intense repetitive stretch-shortening cycles were shown to induce decrements reflex sensitivity [28] and a reduced tolerance to stretch loads [29]. The repetition of stretch-shortening cycles may thus induce a protective strategy to reduce pain from the stressful stretching phase [30].

In MUM, the minimization of musculoskeletal damage is considered a determining factor of performance [31]. Investigating how long-distance runners deal with both severe neuromuscular fatigue and mechanical stress is of interest for both injury prevention and performance. This study investigated (i) the consequences of a 110-km MUM on both tibial shock acceleration and lower leg kinematics; and (ii) the relationship between the amplitude of neuromuscular fatigue and impact shock and kinematics changes post-MUM. We hypothesized that runners would strive to maintain or reduce impact intensity by making kinematic adjustments and that despite these adjustments, impact intensity would increase as a consequence of the severe neuromuscular fatigue impairing cushioning abilities.

## Methods

### Subjects

Twenty-three subjects (13 males, 10 females see Table 1) were recruited after a complete medical examination. All subjects gave written informed consent to participate in this study, which was approved by the local ethical committee (protocol #1208048, Comité de Protection des Personnes Sud-Est 1, France) and in agreement with the Declaration of Helsinki. All subjects were experienced, well-trained MUM runners and free from muscular, bone or joint injuries. They wore their own running shoes and used the same shoe model for all measures (mass: 350 ± 46 g, heel height: 26.9 ± 6.9 mm, heel-forefoot drop: 9.2 ± 2.6 mm).

**Table 1:** Subject characteristics.

	Group (N = 23)	Males (N = 13)	Females (N = 10)
Age (years)	42.7 ± 8.9	41.2 ± 9.3	45.7 ± 5.1
Mass pre-MUM (kg)	67.6 ± 9.8	72.1 ± 6.0	55.8 ± 5.2
Mass post-MUM (kg)	66.1 ± 9.6	70.5 ± 6.1	54.7 ± 4.9
Height (cm)	173 ± 9	178 ± 6	163 ± 3
VO_2max_ (ml·min^-1^·kg^-1^)	58 ± 6	60 ± 5	52 ± 2
Finishing time (hh:mm:ss)	19:35:21 ± 04:00:15	18:10:00 ± 3:13:17	22:16:45 ± 2:57:49

### Experimental design

Two months before the race, subjects came to the laboratory for a medical examination, familiarization to neuromuscular evaluations and a maximal incremental running test. The familiarization to neuromuscular measurements consisted of submaximal and maximal voluntary contractions of KE and PF with and without electrical nerve stimulation. The incremental running test was performed on a treadmill with a 10% slope to identify maximal aerobic speed. The initial speed was set at 4 to 6 km·h^-1^ depending on the performance level of the runner and running speed was increased by 1 km∙h^-1^ every 2.5 min until exhaustion. Respiratory exchange data were collected throughout the running test. The maximal aerobic speed was used to standardize the speed of each subject’s treadmill measurement trials.

The international race supporting the study was the North-Face^®^ Ultra-Trail du Mont-Blanc^®^ 2012 (Chamonix, France). It was a 110-km MUM with total positive elevation change of 5862 m. One or two days before the race start (pre-MUM), subjects completed a running bout and performed KE and PF neuromuscular evaluations. They repeated both biomechanical and neuromuscular testing protocols 57± 21 min after they crossed the finishing line (post-MUM). This delay was due to travel time from the finish line to the laboratory.

### Biomechanical measurements

Pre- and post-MUM measurements were conducted on a level treadmill (Cosmed T170 DE, Delta Medical, Ottignies, Belgium) at a speed corresponding to the subject’s maximal aerobic speed measured on a 10% slope at the familiarization (group: 10.9 ± 1.4 km·h^-1^; females: 10.1 ± 0.7 km·h^-1^; males: 11.5 ± 1.4 km·h^-1^) to assess running kinematics at a relative comfort speed for each subject. Subjects ran for 2 min during which an acquisition was performed for kinematic and acceleration measurements during the last 20 seconds. A mounted uniaxial accelerometer (ADXL150, Analog Devices, Wilmington, USA) was securely fixed with strapping on the distal half of the anteromedial aspect of the right tibia. The acceleration signal was recorded at 1000 Hz using LabChart 7 (ADInstruments, Bella Vista, Australia) and a 12-bit A/D acquisition card (DAS8, National Instruments, Austin, USA). Before being fixed onto the subject’s tibia, the accelerometer was calibrated by using the self-calibration technique recommended by the manufacturer based on the 1 g acceleration of the earth’s gravity. It consists in performing two static 3-sec acquisitions: one with the axis of sensitivity in the vertical plane, so the accelerometer registers a 1 g acceleration, and one with the axis of sensitivity in the horizontal plane, so the accelerometer registers no acceleration. Four retroflective markers were placed on the right leg at the heel, at the fifth metatarsal head on the external face of the shoe and in the sagittal plane at the lateral femoral condyle and lateral malleolus. Once all markers were attached, a ~3-s calibration was performed during which subjects stood straight with feet parallel and arms at the sides. Video data were sampled at 120 Hz using a camera (Basler scA640-120gc, Basler AG, Germany) mounted on a tripod placed ~1.5 m from the treadmill. Marker trajectories were tracked and analyzed in Simi Motion 2D (Simi Reality Motion Systems GmbH, Unterschleissheim, Germany). Accelerometer and video data were systematically calibrated for each subject. Accelerometers and markers were placed at identical locations pre- and post-MUM.

#### Impact-related variables

From a time domain analysis, peak tibial acceleration (PTA) was measured. Then a Fast Fourier Transform was performed on 10 consecutive stance phases recorded during the 20-s acquisition for the frequency domain analysis. The beginning of the stance phase was identified as the deflection before PTA [32]. The end was identified as the local minimum on the tibial acceleration signal. Data sets were padded with zero in order to obtain a total of 2048 data points per acceleration profile. The sampling rate and length of data set analyzed resulted in 0.488-Hz frequency bins. Power and frequency were interpolated so each frequency bin was equal to 1 Hz. The power spectral density (PSD) curve was then extracted and its median frequency over 2 to 100 Hz (MDF) and the PSD of the impact shock region (iPSD), i.e. from 10 to 20 Hz [32], were calculated.

#### Kinematics

**Fig 1:**
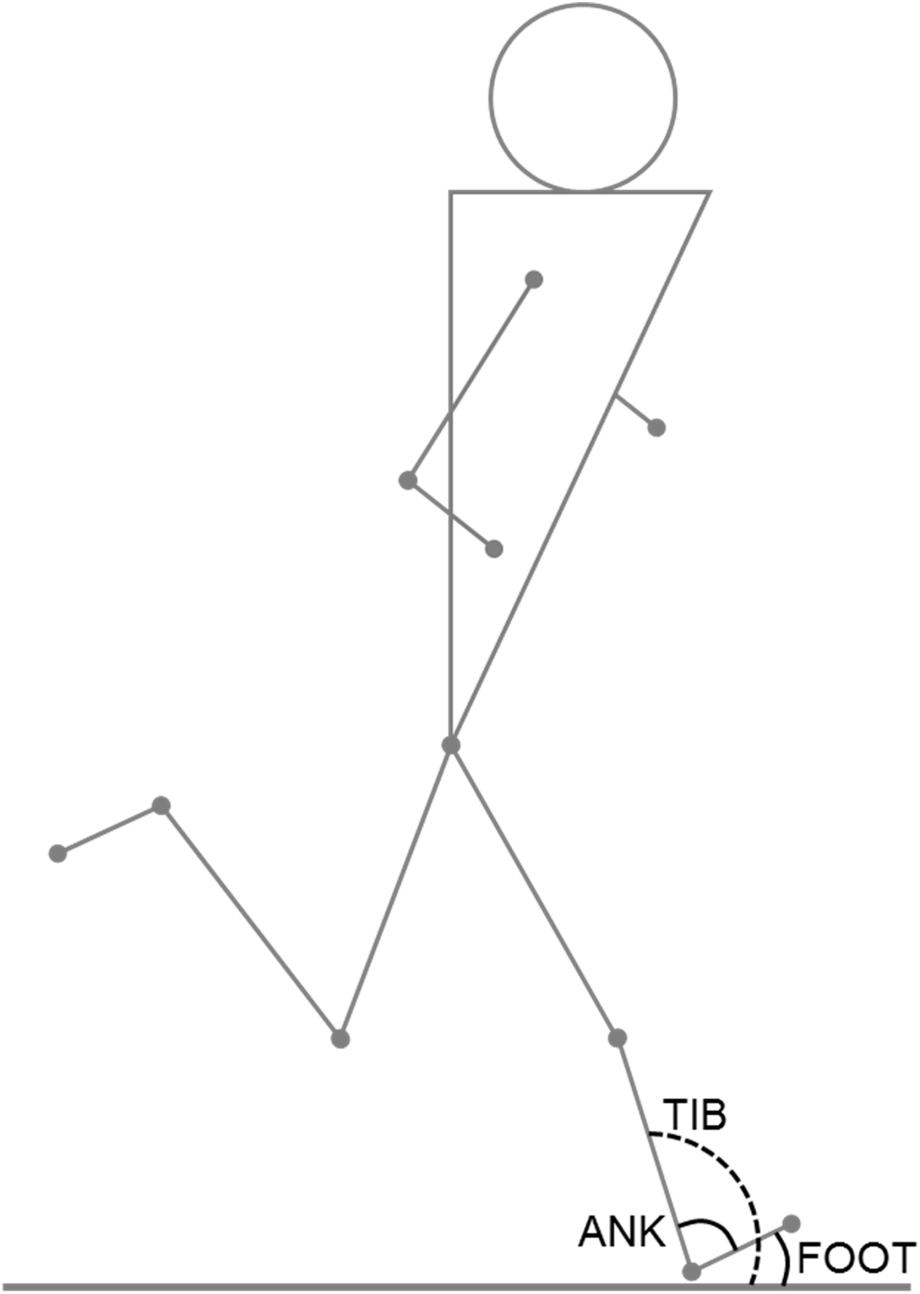
Schematic representation of the kinematic variables measured at initial contact: foot-to-treadmill angle (FOOT), ankle angle (ANK) and tibia-to-treadmill angle (TIB).

Kinematic variables of interest observed at initial contact were foot-to-treadmill angle (FOOT), ankle flexion angle (ANK) and tibia-to-treadmill angle (TIB) (Fig 1). Positive FOOT indicated a rearfoot strike, whereas negative FOOT values indicated a forefoot strike. For ANK, angle values lower than 90° indicated a dorsiflexed ankle. For TIB, angle values greater than 90° indicated a backward tilting of the tibia. Ankle range of motion (ANK_rom_) over each stride cycle was also calculated. Step frequency (SF) was measured from the tibial acceleration signal as the inverse of half stride-cycle duration (i.e. time between two consecutive tibial acceleration peaks).

### Neuromuscular evaluation

Neuromuscular tests were performed for right KE and PF identically pre- and post-MUM as described by Temesi et al. [27]. The neuromuscular tests assessed indices of global, peripheral and central fatigue. For that, KE force and PF torque were measured during voluntary and evoked contractions as explained by Temesi et al. [27].

#### Procedure and torque recording

For both KE and PF, three 5-s MVCs separated by 30 s with electrical nerve stimulation (100-Hz paired pulses and single pulses) delivered at peak torque and immediately after in the relaxed state (100- and 10-Hz paired pulses and single pulses) were performed. Real-time visual feedback was provided and subjects were strongly encouraged during MVCs.

For KE testing, subjects were seated upright in a custom-built chair with both right knee and hips at ~90° of flexion and secured by chest and hips straps. They were asked to keep their arms crossed on their chest during the test. KE force was measured during voluntary and evoked contractions by a calibrated force transducer (Meiri F2732 200 daN, Celians, Montauban, France) with amplifier attached by a non-compliant strap to the right leg just proximal the malleoli of the ankle joint. The force transducer was fixed to the chair such that force was measured in direct line to the applied force.

For PF, subjects were seated upright in a custom-built chair with right ankle, knee and hip joints at ~90° from complete extension. Non-compliant straps secured the chest and hips, and also heel and forefoot to limit heel lift and avoid lateral and frontal displacement. They were also asked to keep their arms crossed on their chest during the test. PF torque was assessed by an instrumented pedal (CS1060 300 Nm, FGP Sensors, Les Clayes Sous Bois, France).

#### Electrical nerve stimulation

Single electrical stimuli of 1-ms duration were delivered via constant-current stimulator (DS7A, Digitimer, Welwyn Garden City, Hertfordshire, UK) to both right femoral and tibial nerves. Surface cathodes (30-mm diameter, Meditrace 100) were manually pressed into the femoral triangle and popliteal fossea, and 50 × 90 mm rectangular anodes (Durastick Plus, DJO Global, Vista, USA) placed in the gluteal fold and over the patellar tendon. To determine the optimal stimulation intensity before neuromuscular testing, single stimuli were delivered incrementally in relaxed muscle until M-wave and twitch amplitudes plateaued for both muscles groups at both pre- and post-MUM. A stimulus intensity of 130% of the intensity to produce maximal M-wave and maximal twitch responses was employed to ensure full spatial recruitment. Intensities of stimulation were 60.8 ± 19.0 mA for KE and 24.9 ± 11.2 mA for PF at pre-MUM and 63.1 ± 19.3 mA for KE and 24.3 ± 10.0 mA for PF at post-MUM.

#### Neuromuscular variables

To describe global fatigue, MVC was calculated as the mean peak force (KE) or torque (PF) from three MVCs. Potentiated peak twitch (TwPot) and high-frequency doublet (Db100) force (KE) or torque (PF) amplitudes were determined as peripheral fatigue indices. The presence of low-frequency fatigue post-MUM was evaluated from the change in the ratio of paired 10- and 100-Hz doublets (10:100). Central fatigue was evaluated by assessing voluntary activation (VA) from both KE and PF as follows:

$$VA=\;[1-\;(Db100_{superimposed}\cdot \;Db100^{-1})]\times 100$$

#### Subjective sensations

Subjects reported general subjective sensation of fatigue and pain in KE and PF on a 100-mm visual analogic scale pre-MUM and immediately upon arrival at the testing site post-MUM.

### Data analysis and statistics

For each subject, percent changes (Δ) from pre- to post-MUM were calculated for kinematic, impact-related and neuromuscular variables, and reported as mean ± standard deviation. Normal distribution was tested by Shapiro-Wilk normality tests and variance homogeneity by Fisher *F*-tests for all variables pre- and post-MUM. Student *t*-tests were performed on pre- and post-MUM kinematic, impact-related and neuromuscular variables, except VA and 10:100 for which Wilcoxon tests were used because normality tests failed for these variables. Bravais-Pearson correlations were computed from the percent changes in kinematic, impact-related and neuromuscular variables. In view of the results obtained from the Student and Wilcoxon tests’, two-way within subjects ANOVAs were computed *a posteriori* to test main effects of FSP and fatigue (TIME) as well as the interaction effect (FSP × TIME) on kinematics and impact-related variables. The significance level was set at *p* < 0.05.

## Results

**Fig 2:**
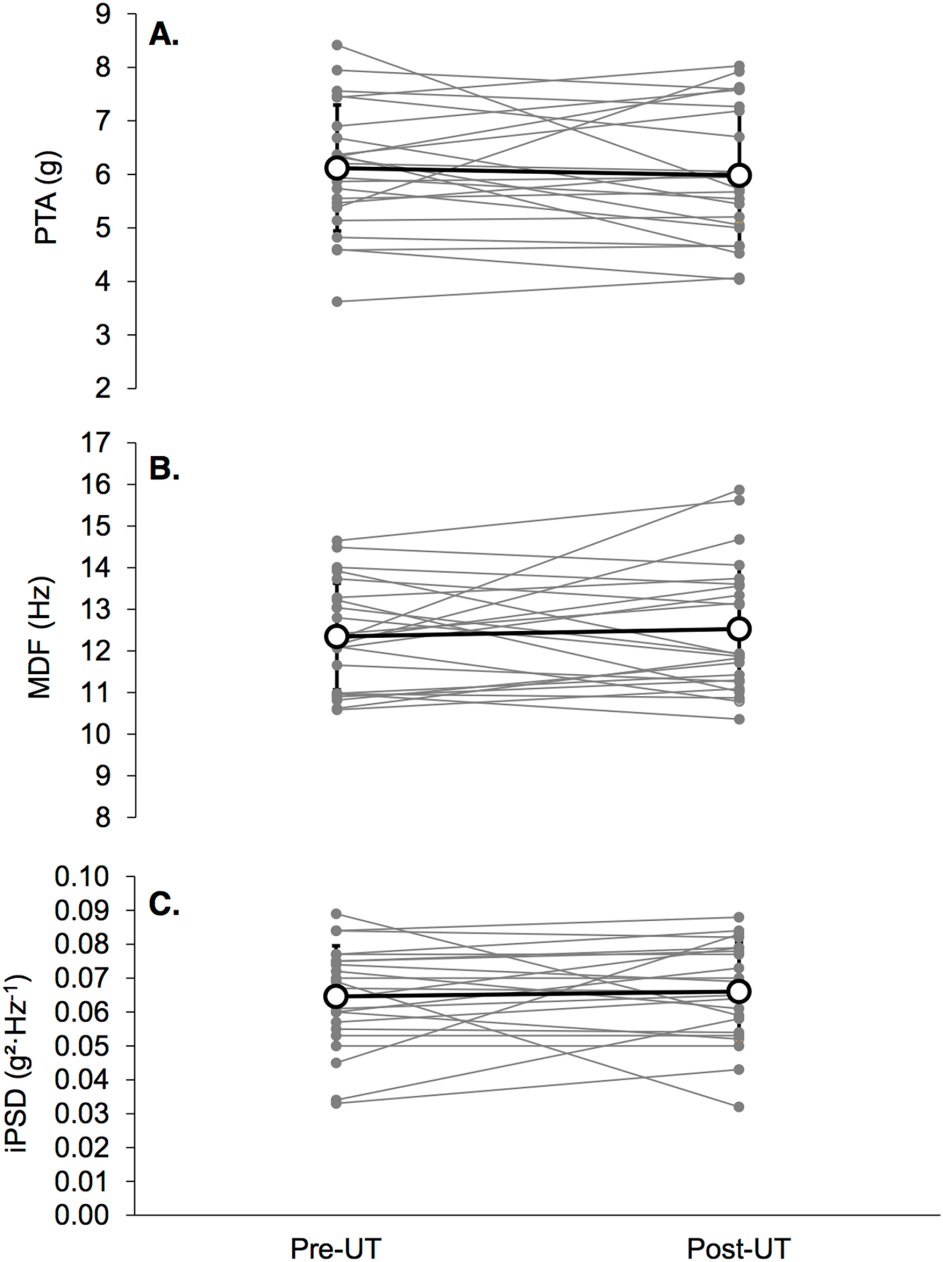
Means (white dots) and standard deviation for impact-related parameters (panel A: PTA, panel B: MDF, panel C: iPSD) pre-MUM and post-MUM, as individual values (gray dots).

**Fig 3:**
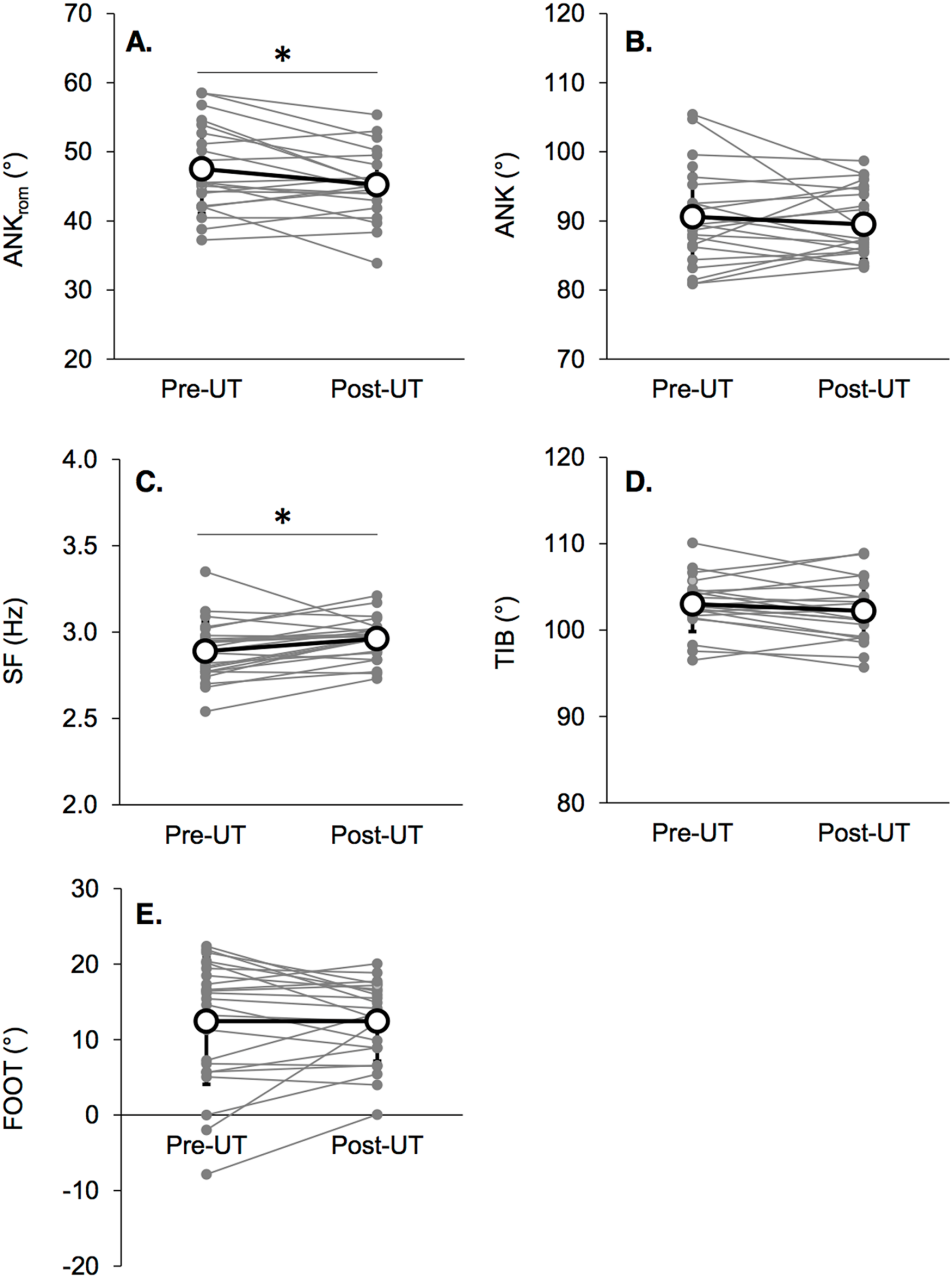
Means (white dots) and standard deviation for kinematic variables (panel A: ANK_rom_, panel B: ANK, panel C: SF, panel D: TIB, panel E: FOOT) pre-MUM and post-MUM, as individual values (gray dots). * denotes significant differences between pre-MUM and post-MUM.

The subjects completed the race in 19:35:21 ± 4:00:15. No significant changes in PTA, MDF or iPSD were found between pre- and post-MUM (Fig 2). Average values of impact-related and kinematic parameters are reported in Tables 2 and 3, respectively. As shown in individual data presented in Fig 1, large inter-subject variability in changes between pre- and post-MUM was found (i.e. PTA: -1.2 ± 17.0%, MDF: 1.8 ± 10.8%, iPSD: 6.3 ± 28.6%). The kinematic parameter SF increased by 2.7 ± 4.1% (*p* = 0.013, Fig 3) and ANK_rom_ decreased by -4.1 ± 8.5% (*p* = 0.024, Fig 3). Other kinematic variables were not altered (ΔFOOT: 0.3 ± 5.3%, ΔANK: -0.2 ± 5.8%, ΔTIB: -0.8 ± 2.2%).

**Table 2:** Mean ± SD for impact-related variables.

	Pre-MUM	Post-MUM
PTA (g)	6.12 ± 1.18	5.98 ± 1.27
MDF (Hz)	12.3 ± 1.28	12.5 ± 1.57
iPSD (g²·Hz^-1^)	0.065 ± 0.015	0.066 ± 0.015

**Table 3:** Mean ± SD for kinematic variables.

	Pre-MUM	Post-MUM
SF (Hz)	2.89 ± 0.17	2.96 ± 0.12*
FOOT (°)	12.4 ± 8.38	12.4 ± 5.29
TIB (°)	102.5 ± 4.02	102.3 ± 3.52
ANK (°)	90.6 ± 7.04	89.5 ± 5.09
ANK_rom_ (°)	47.5 ± 6.44	45.2 ± 4.96*

Significant changes between Pre-MUM and Post-MUM are denoted by * (*p* < 0.05).

**Fig 4:**
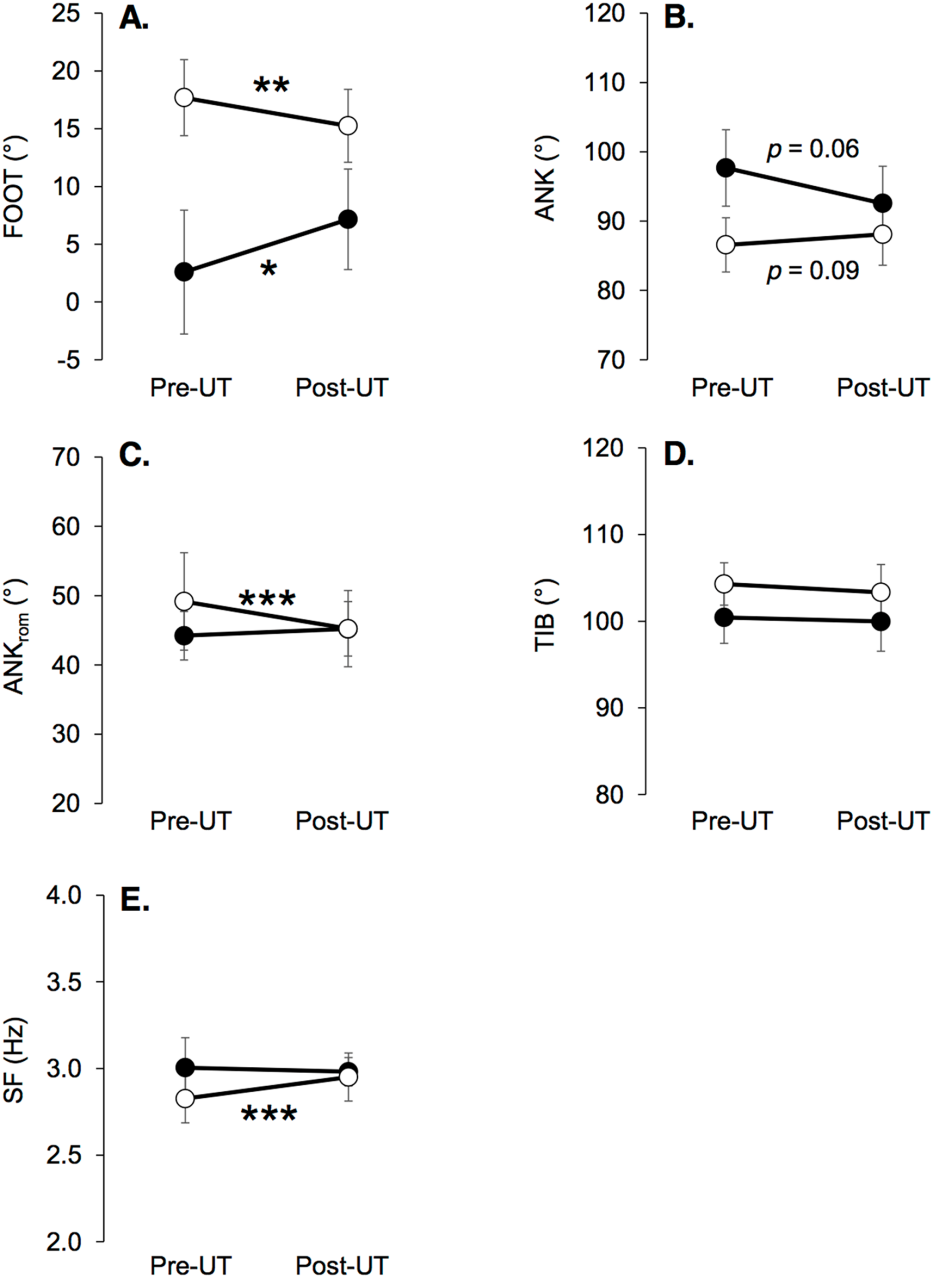
Means and standard deviations for kinematic variables (panel A: FOOT, panel B: ANK, panel C: ANK_rom_, panel D: TIB, panel E: SF) at pre-MUM and post-MUM for rearfoot strikers (n = 15, white dots) and non-rearfoot strikers (n = 8, black dots) as assessed pre-MUM. Significant changes between pre-MUM and post-MUM are denoted by * (*p* < 0.05) or ** (*p* < 0.01).

FOOT ranged from -7.9° to 22.4° pre-MUM, and 0.1° to 20.1° post-MUM, indicating that none of the subjects exhibited a forefoot strike pattern and that runners tended to overall adopt flatter foot landing (Fig 3). However, our statistical results do not support this observation likely because of the large inter-individual variability in running kinematics. Given this observation, we performed *a posteriori* an additional statistical analysis dividing the experimental group in two subgroups based on foot strike pattern (FOOT values) at pre-MUM. Two subgroups were obtained based on the foot strike pattern at pre-MUM according to the criteria defined by Altman and Davis [33] (Fig 4A): a ‘rearfoot strikers (RFS) group’ including subjects with a FOOT value higher than 8° at pre-MUM (n = 15), and a ‘non rearfoot strikers (NRFS) group’ including subjects with a FOOT value lower than 8° (n = 8). Two-way within subjects ANOVAs were computed to test main effects of FSP and fatigue (TIME) as well as the interaction effect (FSP × TIME) on kinematics and impact-related variables. Newman-Keuls post-hoc tests were used to determine differences if the ANOVA revealed a significant main effect. Results of this *a posterior* analysis were presented in Table 4 and Fig 4. As expected, all kinematic variables presented a main FSP effect. For FOOT and ANK, interaction effects were showed. Post-hoc tests revealed that the NRFS group significantly increased FOOT by 5 ± 6% (*p* < 0.001) while decreasing ANK by 5 ± 6% (*p* = 0.039). Conversely, the RFS group tended to decrease FOOT by 2 ± 3% (*p* = 0.091). For SF, the interaction effect tended to be significant (*p* = 0.092).

**Table 4:** Mean ± SD for kinematics and impact-related variables for RFS and NRFS subgroups at Pre-MUM and Post-MUM.

	RFS		NRFS		ANOVA *p* values		
	Pre-MUM	Post-MUM	Pre-MUM	Post-MUM	FSP	TIME	FSP x TIME
FOOT (°)	17.7 ± 3.3^$ $^	15.3 ± 3.2^$ $^	2.6 ± 5.4	7.2 ± 4.4*	**<0.001**	0.379	**0.005**
ANK (°)	86.6 ± 3.9^$ $^	88.1 ± 4.4^$^	97.7 ± 5.5	92.6 ± 5.4*	**<0.001**	0.231	**0.030**
TIB (°)	104.3 ± 2.4	103.3 ± 3.2	100.4 ± 3.0	100.0 ± 3.4	**<0.001**	0.468	0.803
ANK_rom_ (°)	49.2 ± 7.0	45.2 ± 5.5	44.2 ± 3.5	45.2 ± 3.9	0.183	0.429	0.189
SF (Hz)	2.83 ± 0.14	2.95 ± 0.14	3.01 ± 0.17	2.98 ± 0.08	**0.019**	0.249	0.092
PTA (g)	5.82 ± 1.10	5.56 ± 1.02	6.68 ± 1.18	6.77 ± 1.37	**0.005**	0.815	0.621
MDF (Hz)	11.8 ± 0.9	11.9 ± 1.2	13.4 ± 1.2	13.7 ± 1.6	**<0.001**	0.596	0.889
iPSD (g²/Hz)	0.064 ± 0.014	0.061 ± 0.014	0.067 ± 0.017	0.075 ± 0.011	0.062	0.479	0.221

ANOVA *p* values represent the main effects of foot strike pattern (FSP) and fatigue (TIME), and the interaction effect (FSP × TIME). Significant effects were highlighted in bold. Significant differences between Pre-MUM and Post-MUM in the NRFS subgroup were denoted by * (*p* < 0.05). Significant differences between the two subgroups at Pre-MUM or Post-MUM were denoted by ^$^ (*p* < 0.05) or ^$ $^ (*p* < 0.001).

The main central and peripheral fatigue parameters are reported in Table 5. MVCs were significantly decreased post-MUM by 28.2 ± 16.5% (*p* < 0.0001) for PF and 34.7 ± 19.1% (*p* < 0.0001) for KE. Significant deficits in VA were reported for both PF (-11.2 ± 13.8%, *p* < 0.01) and KE (-18.9 ± 13.3%, *p* < 0.0001) post-MUM. KE Db100 and TwPot were reduced by 8.1 ± 19.1% and 11.0 ± 16.2%, respectively (*p* < 0.05). Decreases in PF Db100 (-9.8 ± 13.9%, *p* < 0.05) and TwPot (-16.3 ± 13.5%, *p* < 0.01) were also observed. The presence of low-frequency fatigue was only observed in PF, as indicated by the significant decrease in 10:100 (-5.9 ± 4.9%, *p* < 0.01). As expected, the sensation of global fatigue (pre-MUM: 22.0 ± 16.6 mm, post-MUM: 58.4 ± 18.9 mm), KE pain (pre-MUM: 3.4 ± 7.4 mm, post-MUM: 39.4 ± 28.0 mm) and PF pain (pre-MUM: 2.9 ± 5.9 mm, post-MUM: 52.6 ± 26.0 mm) largely increased after the race (*p* < 0.00001).

**Table 5:** Mean ± SD for neuromuscular variables at Knee Extensors and Plantar Flexors.

	Pre-MUM	Post-MUM
**Knee Extensors**		
MVC (N)	507 ± 142	327 ± 125***
Db100 (N)	234.0 ± 50.4	213.0 ± 52.5*
TwPot (N)	140.0 ± 29.6	124.0 ± 30.5**
10:100 (%)	96.6 ± 10.7	89.9 ± 15.5
VA (%)	92.6 ± 5.6	74.9 ± 12.7***
**Plantar Flexors**		
MVC (Nm)	156 ± 38	112 ± 33***
Db100 (Nm)	44.8 ± 8.3	40.3 ± 8.2*
TwPot (Nm)	29.7 ± 6.1	24.6 ± 5.1**
10:100 (%)	101.0 ± 4.7	95.7 ± 5.3**
VA (%)	97.8 ± 3.4	87.4 ± 13.5**

Significant changes between Pre-MUM and Post-MUM are denoted by * (*p* < 0.05), ** (*p* < 0.01) and *** (*p* < 0.0001). Data previously integrated in Temesi et al. [27].

**Fig 5:**
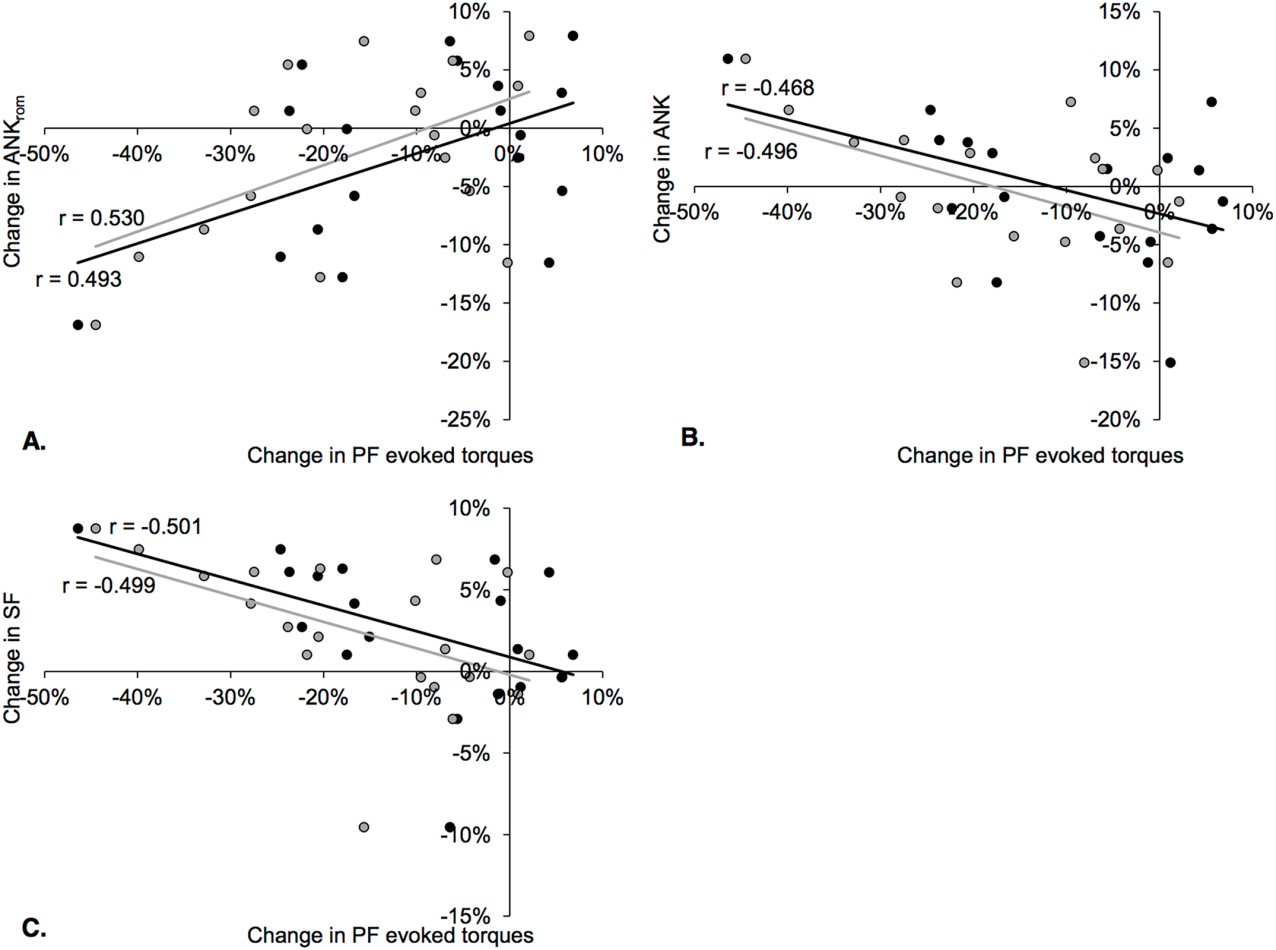
Correlations between percent changes in indicators of PF peripheral fatigue (black dots and linear fit: Db100, gray dots and linear fit: TwPot) and percent changes in kinematic variables (panel A: ANK_rom_, panel B: ANK, panel C: SF).

ΔANK_rom_ was positively correlated with both PF ΔDb100 and PF ΔTwPot (*r* = 0.493, *p* = 0.038, 90% confidence interval (CI) [0.171; 0.720], and *r* = 0.530, *p* = 0.024, CI [0.219; 0.743], respectively, Fig 5A), and it tended to be positively correlated with the absolute change in perceived calf pain between pre- and post-MUM (*r* = 0.426, *p* = 0.054, CI [0.087; 0.677]). ΔANK was negatively correlated with both PF ΔDb100 and PF ΔTwPot (*r* = -0.468, *p* = 0.050, CI [-0.139; -0.704], and *r* = -0.496, *p* = 0.036, CI [-0.174; -0.722], respectively, Fig 5B). Similarly, ΔSF was negatively correlated with both PF ΔDb100 and PF ΔTwPot (*r* = -0.501, *p* = 0.025, CI [-0.181; -0.725], and *r* = -0.499, *p* = 0.025, CI [-0.178; -0.724], respectively, Fig 5C). There were no relationships between the severity of neuromuscular fatigue and changes in impact-related parameters.

## Discussion

The purposes of this study were to investigate the consequences of a 110-km MUM race on intensity and frequency content of tibial shock acceleration and running kinematics, and to determine whether these changes related to neuromuscular fatigue severity. As previously reported [27], the 110-km MUM induced severe neuromuscular alterations, as shown by the decreases in KE and PF MVC (-35% and -28%, respectively) that were both central and peripheral in origin. Increased SF and decreased ANK_rom_ were the only significant kinematic changes post-MUM while changes in impact-related parameters and kinematics were highly variable between individuals. Interestingly, changes in ankle kinematics and SF were correlated with the severity of peripheral PF fatigue.

### Effect of MUM on impact-related parameters and kinematics

No changes in PTA or iPSD were observed after the race. These results contrast previous findings investigating the effects of fatigue induced by ~15-30-min exhaustive running bouts on impact parameters [7,8,9,10,11]. However, the present results agree with those of Abt et al. [14] and Clansey et al. [12] showing no change in PTA after an incremental VO_2max_ treadmill protocol or a 20-min run at lactate threshold, respectively. The discrepancy in fatigue effects on impact may be attributed to protocol design, especially running intensity, but also the extreme race duration. Several authors have suggested that runners would become less tolerant to foot-to-ground impacts in prolonged running, therefore adopting a “smoother and safer running style” [17,18,19,20]. The increased SF post-MUM race agrees with previous studies investigating extreme duration running [17,18,19,20,34] and was the major kinematic adjustment common to all subjects following a MUM race. Morin et al. [19] interpreted this kinematic change as a compensating strategy for decrements in PF propulsive capacity and/or protective behavior from mechanical stress.

### Fatigue-dependent kinematic adjustments

Significant correlations were found between ΔANK_rom_, ΔANK and ΔSF and relative changes in evoked PF forces, i.e. peripheral fatigue (Fig 5). Thus, larger peripheral PF dysfunction, usually interpreting as a decline in force production at the cross-bridge level or excitation-contraction coupling failure [35,36,37], was associated with accentuated increases in SF, increases in plantarflexion at contact, and greater decline in ANK_rom_. A possible explanation for this result could be that high PF muscle fatigue impairs PF contributions during the propulsive phase since they are of prime importance for vertical acceleration permitting flight phases [38,39]. Increased SF has been previously shown to reduce vertical oscillations of the body’s center of mass by increasing leg stiffness [17,40,41]. We thus suggest that runners exhibiting the greatest PF peripheral fatigue may further increase SF because this strategy would be less costly for PF muscles, especially during the propulsive phase. However, runners would not likely switch to an overly pronounced forefoot strike pattern since it would increase negative work at the ankle [42] and PF activity [43,44]. Interestingly, the *a posteriori* statistical analysis described above revealed interaction effects between FSP and fatigue for FOOT and ANK. The NRFS runners before the race adopted at post-MUM a less anterior foot strike pattern after the race whereas runners with RFS before the race tended to adopt a less RFS pattern. These results suggest that ultramarathon runners, whatever their preferred running kinematics (in a non-fatigued state), would tend to adopt an intermediate/midfoot running technique. These kinematic changes were associated to PF fatigue. As previously discussed, they may occur in response to an impairment of the propulsive function of PF, and/or to limit the painful negative work performed by PF during the braking phase (especially when using a NRFS pattern). These changes in running kinematics toward a flatter foot strike pattern do not affect shock magnitude and impact-related vibrations content, i.e. keep constant the impact intensity despite the severe neuromuscular fatigue. It is however worth mentioning that correlations between kinematic changes and the severity of PF fatigue demonstrated large 90% confidence intervals, although significant. Further experiments are needed to confirm these findings.

The greater SF and the lower ANK_rom_, inducing or being induced by the flatter landing, may occur to compensate deficits in muscle function but also in order to accommodate to mechanical strains applied at the musculoskeletal system in response to increased sensitivity to loads applied at bones or joints as protective strategies [12,14]. Indeed, the muscles’ ability to cushion impacts might be impaired by fatigue since they actively participate in shock attenuation [3,4]. Otherwise, correlations observed between the severity of peripheral PF fatigue and either ankle kinematic or SF changes support the paradigm of compensating strategies to fatigue [45,46]. It has been established that compensatory adjustments occur in response to peripheral neuromuscular fatigue. Indeed, the severity of peripheral fatigue is signaled and integrated at a central level *via* increased discharge rates of group III and IV afferents [35]. The activation of these mechanosensitive and metabosensitive afferent fibers may disorganize the sensory-motor loop by the inhibition of motoneurons to reduce activation of damaged muscles [47]. Alternatively, intrafusal fatigue, defined by a decline in muscle-spindle stretch sensitivity induced by muscle damage [28], may be responsible for the kinematic reorganization. Muscle damage and the associated decrease in stretch reflex sensitivity observed after prolonged stretch-shortening exercises [28,29] such as UT races, can likely decrease tolerance to imposed stretch loads and impair limb stiffness regulation, especially muscle stretch-shortening performance [29,48]. Therefore, the kinematic changes observed in the present study can be considered as a way runners compensate for contractile failure and address muscle soreness when performing stretch-shortening cycles.

## Conclusion

The present findings provide further insights into the kinematic adjustments adopted after extremely long running exercises. Whatever their preferred running kinematics before the race, after the race runners tended to adopt a flatter foot strike. The extent of changes in step frequency and ankle kinematics after the race was correlated to the severity of peripheral plantar flexor fatigue: the greater the peripheral fatigue at plantar flexors, the greater the increase in step frequency, the flatter the foot landing, and the greater the decline in the ankle range of motion. These kinematic changes may occur in response to intense musculoskeletal pain that could partly arise from lowered tolerance to muscle stretch loads and/or repetitive shocks as protective adjustments, and/or to counteract muscle contractile failure as compensatory adjustments. Whether fatigue-induced kinematic changes are conscious or unconscious remains unknown and remains to be investigated.

## Supporting Information

S1 Table Means, standard deviations (SD), coefficients of variation (%CV), 95% confidence intervals (95% CI + and 95% CI -) and Cohen’s d coefficients for plantar flexors neuromuscular variables. (DOCX)

S2 Table Means, standard deviations (SD), coefficients of variation (%CV), 95% confidence intervals (95% CI + and 95% CI -) and Cohen’s d coefficients for knee extensors neuromuscular variables. (DOCX)

S3 Table Means, standard deviations (SD), coefficients of variation (%CV), 95% confidence intervals (95% CI + and 95% CI -) and Cohen’s d coefficients for impact-related variables. (DOCX)

S4 Table Means, standard deviations (SD), coefficients of variation (%CV), 95% confidence intervals (95% CI + and 95% CI -) and Cohen’s d coefficients for kinematics. (DOCX)

## References

[1] Radin EL, Parker HG, Pugh JW, Steinberg RS, Paul IL, Rose RM. Response of joints to impact loading. 3. Relationship between trabecular microfractures and cartilage degeneration. J Biomech. 1973;6(1):51–7. Epub 1973/01/01. . 4693868 10.1016/0021-9290(73)90037-7

[2] Valiant GA. Transmission and attenuation of heel strike accelerations In: Cavanagh PR, editor. The biomechanics of distance running. Champaign, IL: Human Kinetics; 1989 p. 225–47.

[3] Boyer KA, Nigg BM. Muscle activity in the leg is tuned in response to impact force characteristics. J Biomech. 2004;37(10):1583–8. Epub 2004/09/01. 10.1016/j.jbiomech.2004.01.002 S0021929004000314 [pii]. . 15336933

[4] Wakeling JM, Nigg BM. Modification of soft tissue vibrations in the leg by muscular activity. J Appl Physiol. 2001;90(2):412–20. Epub 2001/02/13. . 11160036 10.1152/jappl.2001.90.2.412

[5] Horisberger M, Fortuna R, Valderrabano V, Herzog W. Long-term repetitive mechanical loading of the knee joint by in vivo muscle stimulation accelerates cartilage degeneration and increases chondrocyte death in a rabbit model. Clin Biomech. 2013;28(5):536–43. Epub 2013/05/25. 10.1016/j.clinbiomech.2013.04.009 S0268-0033(13)00092-2 [pii]. . 23701865

[6] Warden SJ, Davis IS, Fredericson M. Management and prevention of bone stress injuries in long-distance runners. J Orthop Sports Phys Ther. 2014;44(10):749–65. Epub 2014/08/12. 10.2519/jospt.2014.5334 . 25103133

[7] Derrick TR, Dereu D, McLean SP. Impacts and kinematic adjustments during an exhaustive run. Med Sci Sports Exerc. 2002;34(6):998–1002. Epub 2002/06/06. ***96***. 12048328 10.1097/00005768-200206000-00015

[8] Mizrahi J, Verbitsky O, Isakov E, Daily D. Effect of fatigue on leg kinematics and impact acceleration in long distance running. Hum Movement Sci. 2000;19:139–51. ***92***.

[9] Mizrahi J, Voloshin A, Russek D, Verbistsky O, Isakov E. The influence of fatigue on EMG and impact acceleration in running. Basic Appl Myol. 1997;7(2):111–8. ***102***.

[10] Verbistsky O, Mizrahi J, Voloshin A, Treiger J, Isakov E. Shock transmission and fatigue in human running. J Appl Biomech. 1998;14:300–11. ***105***. 28121250 10.1123/jab.14.3.300

[11] Voloshin AS, Mizrahi J, Verbitsky O, Isakov E. Dynamic loading on the human musculoskeletal system—effect of fatigue. Clin Biomech. 1998;13(7):515–20. Epub 2001/06/21. S0268-0033(98)00030-8 [pii]. ***93***. 11415829 10.1016/s0268-0033(98)00030-8

[12] Clansey AC, Hanlon M, Wallace ES, Lake MJ. Effects of fatigue on running mechanics associated with tibial stress fracture risk. Med Sci Sports Exerc. 2012;44(10):1917–23. Epub 2012/04/25. 10.1249/MSS.0b013e318259480d ***91***. 22525776

[13] Mercer JA, Bates BT, Dufek JS, Hreljac A. Characteristics of shock attenuation during fatigued running. J Sports Sci. 2003;21(11):911–9. Epub 2003/11/25. 10.1080/0264041031000140383 ***104***. 14626370

[14] Abt JP, Sell TC, Chu Y, Lovalekar M, Burdett RG, Lephart SM. Running kinematics and shock absorption do not change after brief exhaustive running. J Strength Cond Res. 2011;25(6):1479–85. Epub 2011/03/10. 10.1519/JSC.0b013e3181ddfcf8 ***19 et 111***. 21386724

[15] Girard O, Millet GP, Slawinski J, Racinais S, Micallef JP. Changes in running mechanics and spring-mass behaviour during a 5-km time trial. Int J Sports Med. 2013;34(9):832–40. Epub 2013/04/04. 10.1055/s-0032-1329958 . 23549688

[16] Rabita G, Couturier A, Dorel S, Hausswirth C, Le Meur Y. Changes in spring-mass behavior and muscle activity during an exhaustive run at VO2max. J Biomech. 2013;46(12):2011–7. Epub 2013/07/16. 10.1016/j.jbiomech.2013.06.011 S0021-9290(13)00275-3 [pii]. . 23850446

[17] Millet GY, Morin JB, Degache F, Edouard P, Feasson L, Verney J, et al Running from Paris to Beijing: biomechanical and physiological consequences. Eur J Appl Physiol. 2009;107(6):731–8. Epub 2009/09/17. 10.1007/s00421-009-1194-3 . 19756701

[18] Degache F, Guex K, Fourchet F, Morin JB, Millet GP, Tomazin K, et al Changes in running mechanics and spring-mass behaviour induced by a 5-hour hilly running bout. J Sports Sci. 2013;31(3):299–304. Epub 2012/10/12. 10.1080/02640414.2012.729136 . 23051041

[19] Morin JB, Samozino P, Millet GY. Changes in running kinematics, kinetics, and spring-mass behavior over a 24-h run. Med Sci Sports Exerc. 2011;43(5):829–36. Epub 2010/10/22. 10.1249/MSS.0b013e3181fec518 . 20962690

[20] Morin JB, Tomazin K, Edouard P, Millet GY. Changes in running mechanics and spring-mass behavior induced by a mountain ultra-marathon race. J Biomech. 2011;44(6):1104–7. 10.1016/j.jbiomech.2011.01.028 21342691

[21] Degache F, Morin JB, Oehen L, Guex K, Giardini G, Schena F, et al Running Mechanics During the World's Most Challenging Mountain Ultra-Marathon. Int J Sports Physiol Perform. 2015 Epub 2015/10/13. 2015–0238 [pii] 10.1123/ijspp.2015-0238 . 26457730

[22] Townshend AD, Worringham CJ, Stewart IB. Spontaneous pacing during overground hill running. Med Sci Sports Exerc. 2010;42(1):160–9. Epub 2009/12/17. 10.1249/MSS.0b013e3181af21e2 . 20010117

[23] Gottschall JS, Kram R. Ground reaction forces during downhill and uphill running. J Biomech. 2005;38(3):445–52. Epub 2005/01/18. S0021929004002283 [pii] 10.1016/j.jbiomech.2004.04.023 ***84***. 15652542

[24] Hamill CL, Clarke TE, Frederick EC, Goodyear LJ, Howley ET. Effects of grade running on kinematics and impact force. Med Sci Sports Exerc. 1984;16:185 ***122***.

[25] Millet GY, Tomazin K, Verges S, Vincent C, Bonnefoy R, Boisson RC, et al Neuromuscular consequences of an extreme mountain ultra-marathon. PLoS One. 2011;6(2):e17059 Epub 2011/03/03. 10.1371/journal.pone.0017059 21364944 PMC3043077

[26] Saugy J, Place N, Millet GY, Degache F, Schena F, Millet GP. Alterations of neuromuscular function after the world's most challenging mountain ultra-marathon. PLoS One. 2013;8(6):e65596 Epub 2013/07/11. 10.1371/journal.pone.0065596 PONE-D-13-02818 [pii]. 23840345 PMC3694082

[27] Temesi J, Arnal PJ, Rupp T, Feasson L, Cartier R, Gergele L, et al Are Females More Resistant to Extreme Neuromuscular Fatigue? Med Sci Sports Exerc. 2015;47(7):1372–82. Epub 2014/10/12. 10.1249/MSS.0000000000000540 . 25304334

[28] Avela J, Kyrolainen H, Komi PV, Rama D. Reduced reflex sensitivity persists several days after long-lasting stretch-shortening cycle exercise. J Appl Physiol. 1999;86(4):1292–300. Epub 1999/04/08. . 10194215 10.1152/jappl.1999.86.4.1292

[29] Nicol C, Komi PV, Marconnet P. Fatigue effects of marathon running on neuromuscular performance. Scand J Med Sci Sports. 1991;1(1):10–7.

[30] Morio C, Nicol C, Barla C, Barthelemy J, Berton E. Acute and 2 days delayed effects of exhaustive stretch-shortening cycle exercise on barefoot walking and running patterns. Eur J Appl Physiol. 2012;112(8):2817–27. Epub 2011/11/30. 10.1007/s00421-011-2242-3 . 22124522

[31] Millet GY, Hoffman MD, Morin JB. Sacrificing economy to improve running performance—a reality in the ultramarathon? J Appl Physiol. 2012;113(3):507–9. Epub 2012/04/12. 10.1152/japplphysiol.00016.2012 japplphysiol.00016.2012 [pii]. . 22492933

[32] Shorten MR, Winslow DS. Spectral analysis of impact shock during running. Int J Sports Biomechanics. 1992;8:288–304.

[33] Altman AR, Davis IS. A kinematic method for footstrike pattern detection in barefoot and shod runners. Gait Posture. 2012;35(2):298–300. Epub 2011/11/15. 10.1016/j.gaitpost.2011.09.104 S0966-6362(11)00401-2 [pii]. 22075193 PMC3278526

[34] Vernillo G, Savoldelli A, Zignoli A, Skafidas S, Fornasiero A, La Torre A, et al Energy cost and kinematics of level, uphill and downhill running: fatigue-induced changes after a mountain ultramarathon. J Sports Sci. 2015:1–8. Epub 2015/03/10. 10.1080/02640414.2015.1022870 . 25751128

[35] Gandevia SC. Spinal and supraspinal factors in human muscle fatigue. Physiol Rev. 2001;81(4):1725–89. Epub 2001/10/03. . 11581501 10.1152/physrev.2001.81.4.1725

[36] Millet GY, Martin V, Martin A, Verges S. Electrical stimulation for testing neuromuscular function: from sport to pathology. Eur J Appl Physiol. 2011;111(10):2489–500. Epub 2011/05/19. 10.1007/s00421-011-1996-y . 21590274

[37] Place N, Yamada T, Bruton JD, Westerblad H. Muscle fatigue: from observations in humans to underlying mechanisms studied in intact single muscle fibres. Eur J Appl Physiol. 2010;110(1):1–15. Epub 2010/04/27. 10.1007/s00421-010-1480-0 . 20419312

[38] Sasaki K, Neptune RR. Individual muscle contributions to the axial knee joint contact force during normal walking. J Biomech. 2010;43(14):2780–4. Epub 2010/07/27. 10.1016/j.jbiomech.2010.06.011 S0021-9290(10)00347-7 [pii]. 20655046 PMC2963724

[39] Ellis RG, Sumner BJ, Kram R. Muscle contributions to propulsion and braking during walking and running: insight from external force perturbations. Gait Posture. 2014;40(4):594–9. Epub 2014/08/07. 10.1016/j.gaitpost.2014.07.002 S0966-6362(14)00618-3 [pii]. . 25096545

[40] Farley CT, Gonzalez O. Leg stiffness and stride frequency in human running. J Biomech. 1996;29(2):181–6. Epub 1996/02/01. 0021929095000291 [pii]. . 8849811 10.1016/0021-9290(95)00029-1

[41] Morin JB, Samozino P, Zameziati K, Belli A. Effects of altered stride frequency and contact time on leg-spring behavior in human running. J Biomech. 2007;40(15):3341–8. Epub 2007/07/03. S0021-9290(07)00203-5 [pii] 10.1016/j.jbiomech.2007.05.001 . 17602692

[42] Hamill J, Gruber AH, Derrick TR. Lower extremity joint stiffness characteristics during running with different footfall patterns. Eur J Sport Sci. 2014;14(2):130–6. Epub 2014/02/19. 10.1080/17461391.2012.728249 . 24533519

[43] Giandolini M, Arnal PJ, Millet GY, Peyrot N, Samozino P, Dubois B, et al Impact reduction during running: efficiency of simple acute interventions in recreational runners. Eur J Appl Physiol. 2013;113(3):599–609. Epub 2012/08/10. 10.1007/s00421-012-2465-y . 22875194

[44] Shih Y, Lin KL, Shiang TY. Is the foot striking pattern more important than barefoot or shod conditions in running? Gait Posture. 2013 Epub 2013/03/20. S0966-6362(13)00117-3 [pii] 10.1016/j.gaitpost.2013.01.030 ***124***. 23507028

[45] Forestier N, Nougier V. The effects of muscular fatigue on the coordination of a multijoint movement in human. Neurosci Lett. 1998;252(3):187–90. Epub 1998/09/18. S0304-3940(98)00584-9 [pii]. . 9739992 10.1016/s0304-3940(98)00584-9

[46] Huffenus AF, Amarantini D, Forestier N. Effects of distal and proximal arm muscles fatigue on multi-joint movement organization. Exp Brain Res. 2006;170(4):438–47. Epub 2005/12/22. 10.1007/s00221-005-0227-3 . 16369793

[47] Marqueste T, Decherchi P, Messan F, Kipson N, Grelot L, Jammes Y. Eccentric exercise alters muscle sensory motor control through the release of inflammatory mediators. Brain Res. 2004;1023(2):222–30. Epub 2004/09/18. 10.1016/j.brainres.2004.07.027 S0006-8993(04)01143-6 [pii]. . 15374748

[48] Gollhofer A, Komi PV, Miyashita M, Aura O. Fatigue during stretch-shortening cycle exercises: changes in mechanical performance of human skeletal muscle. Int J Sports Med. 1987;8(2):71–8. Epub 1987/04/01. 10.1055/s-2008-1025644 . 3596879

