# Supplementary material for: Effect of the Fatigue Induced by a 110-km Ultramarathon on Tibial Impact Acceleration and Lower Leg Kinematics
Source: PLoS One. 2016 Mar 31;11(3):e0151687. doi: 10.1371/journal.pone.0151687 (PMC4816299; doi:10.1371/journal.pone.0151687)
Supplement: S1 Table — (DOCX) [file pone.0151687.s001.docx]

**Supplement File 1.** Means, standard deviations (SD), coefficients of variation (%CV), 95% confidence intervals (95% CI + and 95% CI -) and Cohen’s d coefficients for plantar flexors neuromuscular variables.

|  | PLANTAR FLEXORS | | | | | | | | | | | | | | | |
| --- | --- | --- | --- | --- | --- | --- | --- | --- | --- | --- | --- | --- | --- | --- | --- | --- |
|  | MVC | | | Db 100Hz | | | Tw | | | 10:100 | | | %AV | | | |
|  | Pre | Post | %Pre-Post | Pre | Post | %Pre-Post | Pre | Post | %Pre-Post | Pre | Post | %Pre-Post | Pre | Post | %Pre-Post |  |
| Mean | 156.2 | 111.9 | -28.2% | 44.8 | 40.3 | -9.8% | 29.7 | 24.5 | -16.3% | 101.8 | 95.7 | -5.9% | 97.8 | 87.4 | -11.2% |  |
| SD | 38.4 | 33.7 | 16.5% | 8.3 | 8.2 | 13.9% | 6.1 | 5.1 | 13.5% | 4.7 | 5.3 | 4.9% | 3.4 | 13.5 | 13.8% |  |
| %CV | 24.6% | 30.1% | -58.4% | 18.4% | 20.3% | -141.2% | 20.6% | 21.0% | -83.0% | 4.6% | 5.5% | -83.4% | 3.4% | 15.4% | -123.9% |  |
| 95% CI + | 159.6 | 115.0 | -26.7% | 45.6 | 41.1 | -8.5% | 30.3 | 25.1 | -14.9% | 102.2 | 96.2 | -5.4% | 98.1 | 88.7 | -9.8% |  |
| 95% CI - | 152.8 | 108.7 | -29.8% | 44.0 | 39.5 | -11.2% | 29.1 | 24.0 | -17.6% | 101.3 | 95.2 | -6.4% | 97.5 | 86.0 | -12.5% |  |
| Cohen's d (Pre-Post) | 1.18 |  |  | 0.59 |  |  | 0.85 |  |  | 1.30 |  |  | 3.29 |  |  |  |
