# Supplementary material for: Effect of the Fatigue Induced by a 110-km Ultramarathon on Tibial Impact Acceleration and Lower Leg Kinematics
Source: PLoS One. 2016 Mar 31;11(3):e0151687. doi: 10.1371/journal.pone.0151687 (PMC4816299; doi:10.1371/journal.pone.0151687)
Supplement: S2 Table — (DOCX) [file pone.0151687.s002.docx]

**Supplement File 2.** Means, standard deviations (SD), coefficients of variation (%CV), 95% confidence intervals (95% CI + and 95% CI -) and Cohen’s d coefficients for knee extensors neuromuscular variables.

|  | KNEE EXTENSORS | | | | | | | | | | | | | | | | | |
| --- | --- | --- | --- | --- | --- | --- | --- | --- | --- | --- | --- | --- | --- | --- | --- | --- | --- | --- |
|  | MVC | | | Db 100Hz | | | Tw | | | | 10:100 | | | | %AV | | | |
|  | Pre | Post | %Pre-Post | Pre | Post | %Pre-Post | Pre | Post | %Pre-Post | Pre | | Post | %Pre-Post | Pre | | Post | %Pre-Post |  |
| Mean | 506.7 | 326.7 | -34.7% | 233.8 | 212.5 | -8.1% | 139.9 | 123.5 | -11.0% | 96.6 | | 89.9 | -6.4% | 92.6 | | 74.9 | -18.9% |  |
| SD | 141.8 | 124.5 | 19.1% | 50.4 | 52.5 | 19.1% | 29.6 | 30.5 | 16.1% | 10.7 | | 15.5 | 14.7% | 5.6 | | 12.6 | 13.3% |  |
| %CV | 28.0% | 38.1% | -54.9% | 21.6% | 24.7% | -237.2% | 21.2% | 24.7% | -147.1% | 11.1% | | 17.2% | -228.0% | 6.0% | | 16.9% | -70.4% |  |
| 95% CI + | 520.6 | 338.9 | -32.9% | 238.7 | 217.6 | -6.2% | 142.6 | 126.3 | -9.5% | 97.6 | | 91.3 | -5.1% | 93.1 | | 76.2 | -17.6% |  |
| 95% CI - | 492.8 | 314.5 | -36.6% | 228.9 | 207.3 | -9.9% | 137.1 | 120.6 | -12.5% | 95.6 | | 88.4 | -7.8% | 92.0 | | 73.6 | -20.3% |  |
| Cohen's d (Pre-Post) | 1.27 |  |  | 0.42 |  |  | 0.55 |  |  | 0.63 | |  |  | 3.16 | |  |  |  |
