# Supplementary material for: Effect of the Fatigue Induced by a 110-km Ultramarathon on Tibial Impact Acceleration and Lower Leg Kinematics
Source: PLoS One. 2016 Mar 31;11(3):e0151687. doi: 10.1371/journal.pone.0151687 (PMC4816299; doi:10.1371/journal.pone.0151687)
Supplement: S3 Table — (DOCX) [file pone.0151687.s003.docx]

**Supplement File 3.** Means, standard deviations (SD), coefficients of variation (%CV), 95% confidence intervals (95% CI + and 95% CI -) and Cohen’s d coefficients for impact-related variables.

|  | IMPACT | | | | | | | | |
| --- | --- | --- | --- | --- | --- | --- | --- | --- | --- |
|  | PTA |  |  | MDF |  |  | iPSD |  |  |
|  | Pre | Post | %Pre-Post | Pre | Post | %Pre-Post | Pre | Post | %Pre-Post |
| Mean | 6.12 | 5.98 | -1.2% | 12.3 | 12.5 | 1.8% | 0.065 | 0.066 | 6.3% |
| SD | 1.18 | 1.27 | 17.0% | 1.3 | 1.6 | 10.8% | 0.015 | 0.014 | 28.6% |
| %CV | 19.2% | 21.2% | -1418.4% | 10.4% | 12.5% | 602.0% | 23.2% | 21.9% | 456.3% |
| 95% CI + | 6.22 | 6.09 | 0.2% | 12.5 | 12.7 | 2.7% | 0.066 | 0.067 | 8.7% |
| 95% CI - | 6.02 | 5.87 | -2.6% | 12.2 | 12.4 | 0.9% | 0.063 | 0.065 | 3.8% |
| Cohen's d (Pre-Post) | 0.12 |  |  | 0.14 |  |  | 0.10 |  |  |
