# Supplementary material for: Effect of the Fatigue Induced by a 110-km Ultramarathon on Tibial Impact Acceleration and Lower Leg Kinematics
Source: PLoS One. 2016 Mar 31;11(3):e0151687. doi: 10.1371/journal.pone.0151687 (PMC4816299; doi:10.1371/journal.pone.0151687)
Supplement: S4 Table — (DOCX) [file pone.0151687.s004.docx]

**Supplement File 4.** Means, standard deviations (SD), coefficients of variation (%CV), 95% confidence intervals (95% CI + and 95% CI -) and Cohen’s d coefficients for kinematics.

|  | KINEMATICS | | | | | | | | | | | | | | | |
| --- | --- | --- | --- | --- | --- | --- | --- | --- | --- | --- | --- | --- | --- | --- | --- | --- |
|  | FOOT | | | ANK | | | TIB | | | SF | | | ANK rom | | | |
|  | Pre | Post | %Pre-Post | Pre | Post | %Pre-Post | Pre | Post | %Pre-Post | Pre | Post | %Pre-Post | Pre | Post | %Pre-Post |  |
| Mean | 12.4 | 12.4 | 0.3% | 90.6 | 89.5 | -0.2% | 103.0 | 102.2 | -0.8% | 2.89 | 2.96 | 2.7% | 47.5 | 45.2 | -4.1% |  |
| SD | 8.4 | 5.3 | 5.3% | 7.0 | 5.1 | 5.8% | 3.2 | 3.6 | 2.2% | 0.17 | 0.12 | 4.2% | 6.4 | 5.0 | 8.5% |  |
| %CV | 67.3% | 42.5% | 1602.9% | 7.8% | 5.7% | -2505.8% | 3.1% | 3.5% | -285.5% | 6.0% | 4.1% | 154.7% | 13.6% | 11.0% | -210.0% |  |
| 95% CI + | 13.2 | 12.9 | 0.8% | 91.2 | 90.0 | 0.3% | 103.3 | 102.5 | -0.6% | 2.90 | 2.97 | 3.0% | 48.1 | 45.7 | -3.3% |  |
| 95% CI - | 11.7 | 12.0 | -0.1% | 90.0 | 89.1 | -0.8% | 102.7 | 101.9 | -1.0% | 2.87 | 2.95 | 2.3% | 46.9 | 44.8 | -4.9% |  |
| Cohen's d (Pre-Post) | <0.01 |  |  | 0.07 |  |  | 0.26 |  |  | 0.42 |  |  | 0.35 |  |  |  |
